# LncRNA SLCO4A1-AS1 promotes colorectal cancer cell proliferation by enhancing autophagy via miR-508-3p/PARD3 axis

**DOI:** 10.18632/aging.102081

**Published:** 2019-07-16

**Authors:** Zhaozhi Wang, Jianjun Jin

**Affiliations:** 1 Department of Gastrointestinal Medicine, The First Affiliated Hospital and College of Clinical Medicine of Henan University of Science and Technology, Luoyang 471003, Henan, China

**Keywords:** colorectal cancer (CRC), SLCO4A1-AS1, PARD3, miR-508-3p, autophagy

## Abstract

Aberrant expressions of various long non-coding RNAs (lncRNAs) have been involved in the progression and pathogenesis of various carcinomas. However, the expression and biological function of SLCO4A1-AS1 in colorectal cancer (CRC) remain poorly understood. Gain- and loss-of-function assays were applied to determine the roles of SLCO4A1-AS1 in autophagy and CRC progression. qRT-PCR and in situ hybridization (ISH) results showed that SLCO4A1-AS1 was positively associated with PARD3 expression in CRC tissues. *In vitro* and *in vivo* studies revealed that SLCO4A1-AS1 knockdown repressed cytoprotective autophagy as assayed by transmission electron microscopy (TEM), and inhibited cell proliferation by directly targeting partition-defective 3 (PARD3). Mechanistically, SLCO4A1-AS1 acted as a sponge of miR-508-3p, leading to upregulation of PARD3 and promotion of CRC cell proliferation. The current study demonstrates that the SLCO4A1-AS1/miR-508-3p/PARD3/autophagy pathway play a critical role in CRC cell proliferation, and might provide novel targets for developing therapeutic strategies for CRC.

## INTRODUCTION

Colorectal cancer (CRC) is one of the most common cancer types worldwide, with roughly 1.2 million newly diagnosed cases and 60,000 fatalities per year. Due to the development of novel and effective drugs for preventing and combating the disease, CRC morbidity and mortality are declining [1–5]. However, CRC has low overall 5-year survival rate because most patients develop metastatic CRC at the time of diagnosis [6, 7]. Therefore, developing new molecular therapies to improve the therapeutic outcome of CRC is urgently needed.

Long non-coding RNAs (LncRNAs) are non-coding nucleotide transcripts longer than 200 nt, which are involved in various pathophysiological and biological processes, especially in the occurrence and development of tumors [8]. For instance, lncRNA DUXAP8 augments the cell cycle progression of renal cell carcinoma [9]. LncRNA LINC00152 acts as an oncogene to promote the proliferation and metastasis of oral squamous cell carcinoma [10]. LncRNA MNX1-AS1 increases the proliferation, migration, and invasion of cervical cancer cells [11]. LncRNA CALML3-AS1 has been reported to regulate miR-4316/ZBTB2 pathway in bladder cancer development [12]. Nevertheless, the functional role and the underlying mechanism of SLCO4A1-AS1 in CRC remain unclear.

In this study, we found that SLCO4A1-AS1 level was positively correlated with PARD3 expression in CRC patients, and PARD3 protein was the key molecule to trigger the initiation of autophagy. SLCO4A1-AS1 promoted autophagy and growth of CRC cells both *in vitro* and *in vivo*. Furthermore, we found that SLCO4A1-AS1 regulated miR-508-3p expression by acting as a miRNA sponge to regulate PARD3. Taken together, our study demonstrated that the SLCO4A1-AS1/miR-508-3p/PARD3/autophagy signaling modulated the CRC cell proliferation, and might provide novel targets for developing CRC therapeutic strategies.

## RESULTS

### Expression of PARD3 protein levels were positively associated with SLCO4A1-AS1 in human CRC tissues

**Figure 1:**
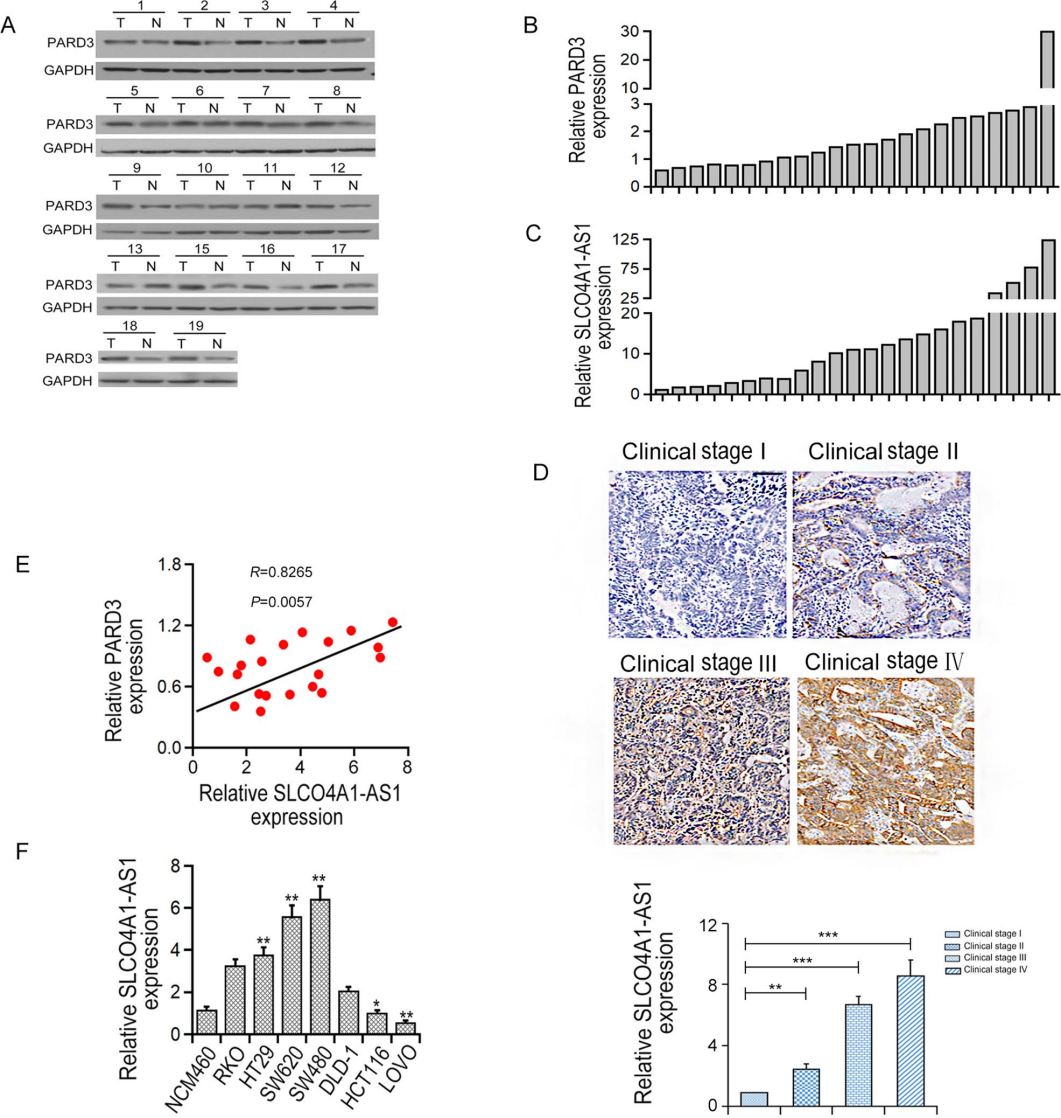
**PARD3 protein level parallels to SLCO4A1-AS1 in human CRC tissues.** (**A**, **B**): Expression of PARD3 protein in CRC tissues (T) (n=23) and the corresponding adjacent control specimens (**C**) (n=23) by Western blot analysis. (**C**) PARD3 protein expression was up-regulated in 23 CRC tissues. (**D**) SLCO4A1-AS1 was elevated gradually with advanced tumor stage (stage I-IV) by in situ hybridization assay; (**E**) The mRNA expression level of SLCO4A1-AS1 was positively correlated with PARD3 protein in CRC tissues (n=23, R = 0.8265, P = 0.0057); (**F**) The level of SLCO4A1-AS1 mRNA in CRC cell lines by qRT-PCR. * P < 0.05, **P < 0.01

As shown in Figure 1A–1B, PARD3 protein was overexpressed in most CRC tissues (19 cases out of 23) compared with adjacent control tissues. In addition, the expression of SLCO4A1-AS1 was significantly up-regulated in 23 CRC tissues compared with the control tissues (Figure 1C). ISH analysis illustrated that SLCO4A1-AS1 levels were elevated in higher clinical stage of CRC (P<0.001, Figure 1D). Correlation analysis indicated that PARD3 protein was positively correlated with the SLCO4A1-AS1 expression in CRC tissues (r=0.8265, P<0.01, Figure 1E). Moreover, expression levels of SLCO4A1-AS1 in SW620, SW480, HT29, DLD-1, and RKO CRC cell lines were significantly up-regulated compared with the normal intestinal epithelium cell line NCM460 (Figure 1F). These results illustrated that the expression of SLCO4A1-AS1 was positively correlated with PARD3 protein level in CRC.

### SLCO4A1-AS1 induced autophagy via activatingPARD3 signaling

**Figure 2:**
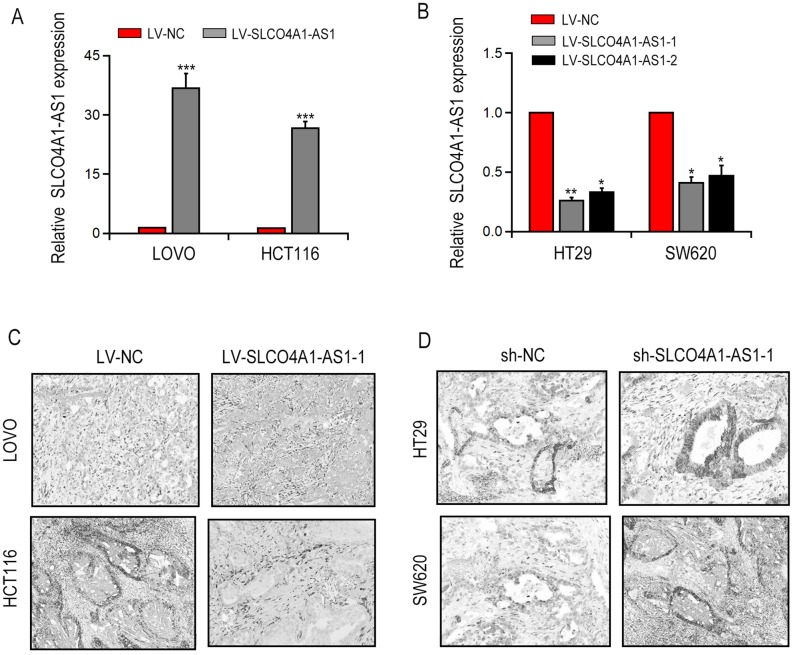
**SLCO4A1-AS1 knockdown decreased autophagy in CRC cells.** (**A**) Overexpressed SLCO4A1-AS1 was found after specific lentivirus transfection in LOVO and HCT116 cells; (**B**) SLCO4A1-AS1 was remarkedly suppressed after sh-SLCO4A1-AS1 transfection in HT29 and SW620 cells; (**C**, **D**) Increased or decreased autophagic vacuole numbers were found after SLCO4A1-AS1 overexpression or knockdown in LOVO and HCT116 cells by TEM. * P < 0.05, **P < 0.01 and ***P < 0.001.

**Figure 3:**
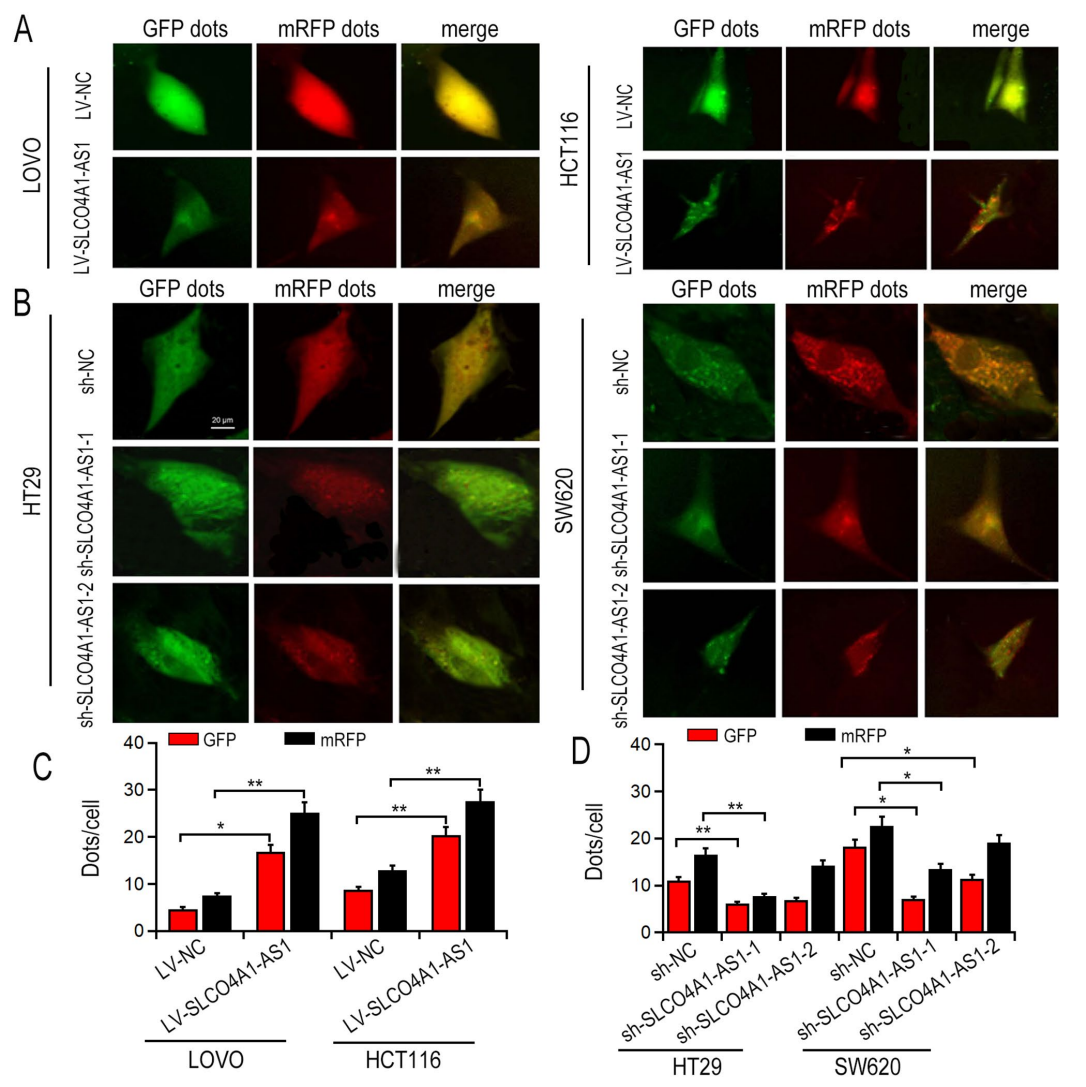
**GFP-LC3 dots detection after SLCO4A1-AS1 overexpression or knockdown.** (**A**, **B**) Representative images in SLCO4A1-AS1-overexpressed or knockdown cells. (**C**, **D**) Quantitation of GFP-LC3 puncta in SLCO4A1-AS1-overexpressed or knockdown cells. * P < 0.05 and **P < 0.01.

**Figure 4:**
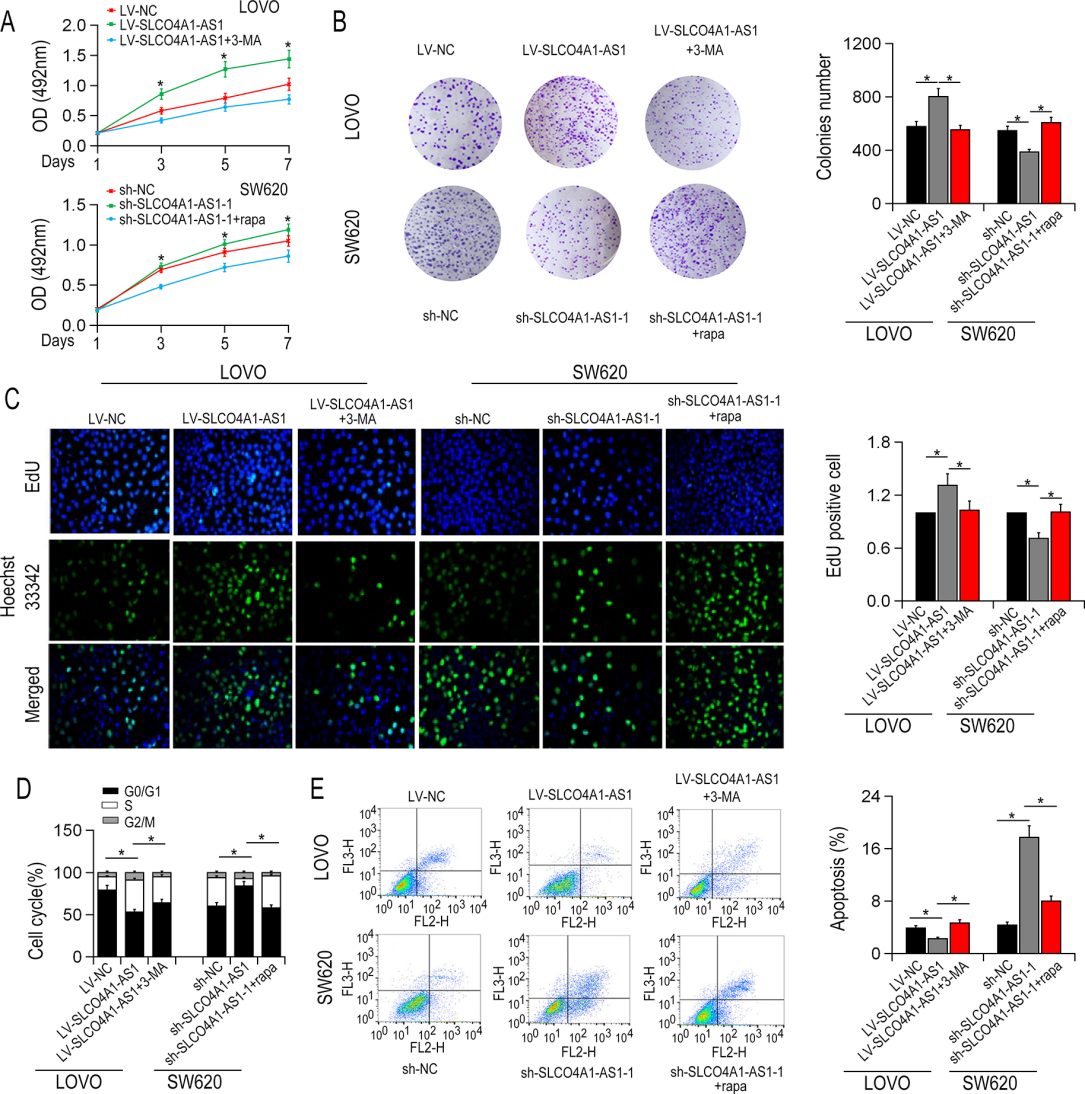
**SLCO4A1-AS1 overexpression promoted CRC cell proliferation.** (**A**) Up-regulation of SLCO4A1-AS1 promoted cell proliferation in LOVO cells by MTS assay. (**B**) SLCO4A1-AS1 overexpression enhanced cell proliferative rate in LOVO cells detected by colony formation assay. (**C**) EdU incorporation assays indicated the cell proliferation rate after SLCO4A1-AS1 overexpression or knockdown; (**D**) The ratio of S phase cells increased after overexpression of SLCO4A1-AS1 in LOVO cells. SLCO4A1-AS1 knockdown repressed S phase SW620 cells. (**E**) Apoptotic rate decreased after up-regulation of SLCO4A1-AS1 in LOVO cells. *P < 0.05

First, we transfected the full-length SLCO4A1-AS1 cDNA or SLCO4A1-AS1-shRNA into LOVO and HCT116 cells to make SLCO4A1-AS1 ectopically expressed in the cells. qRT-PCR results showed that SLCO4A1-AS1 overexpression (Figure 2A) or knockdown (Figure 2B) was observed. Transmission electron microscopy (TEM) analysis revealed that overexpression of SLCO4A1-AS1 enhanced the cytolysosome number in LOVO and HCT116 cells (Figure 2C). In contrast, the cytolysosome number was reduced after SLCO4A1-AS1 knockdown in HT29 and SW620 cells (Figure 2D). Consistent with the findings of TEM, SLCO4A1-AS1 overexpression increased the number of green fluorescent LC3 (Figure 3A, 3C), and down-regulation of SLCO4A1-AS1 expression reduced the number of green fluorescent LC3 (Figure 3B, 3D), which indicated that SLCO4A1-AS1 is an inducer of autophagy in CRC cells. As illustrated in Figure 4A, overexpression of SLCO4A1-AS1 significantly increased cell growth, whereas treatment with autophagy inhibitor 3-methylladenine (3-MA) blocked this effect of SLCO4A1-AS1. Similarly, colony formation assay showed that SLCO4A1-AS1 overexpression increased the colony number in CRC cells, which was decreased by 3-MA treatment. In addition, treatment with autophagy inducer rapamycin restored the proliferation rate of CRC cells suppressed by SLCO4A1-AS1 knockdown (Figure 4B). Furthermore, EdU positive cells at mitosis S-stage increased with the overexpression of SLCO4A1-AS1 in LOVO cells, and the number of EdU-positive cells was decreased after 3-MA treatment (Figure 4C). On the contrary, SLCO4A1-AS1 knockdown decreased the number of EdU-positive cells, which was restored by treatment with rapamycin (Figure 4C). Flow cytometry assay showed that cell cycle distribution was consistent with that result from EdU staining (Figure 4D). Moreover, SLCO4A1-AS1 overexpression repressed cell apoptosis, whereas this effect was blocked by autophagy inhibition (Figure 4E). In conclusion, the data suggested that SLCO4A1-AS1 may induce protective autophagy in CRC cells.

**Figure 5:**
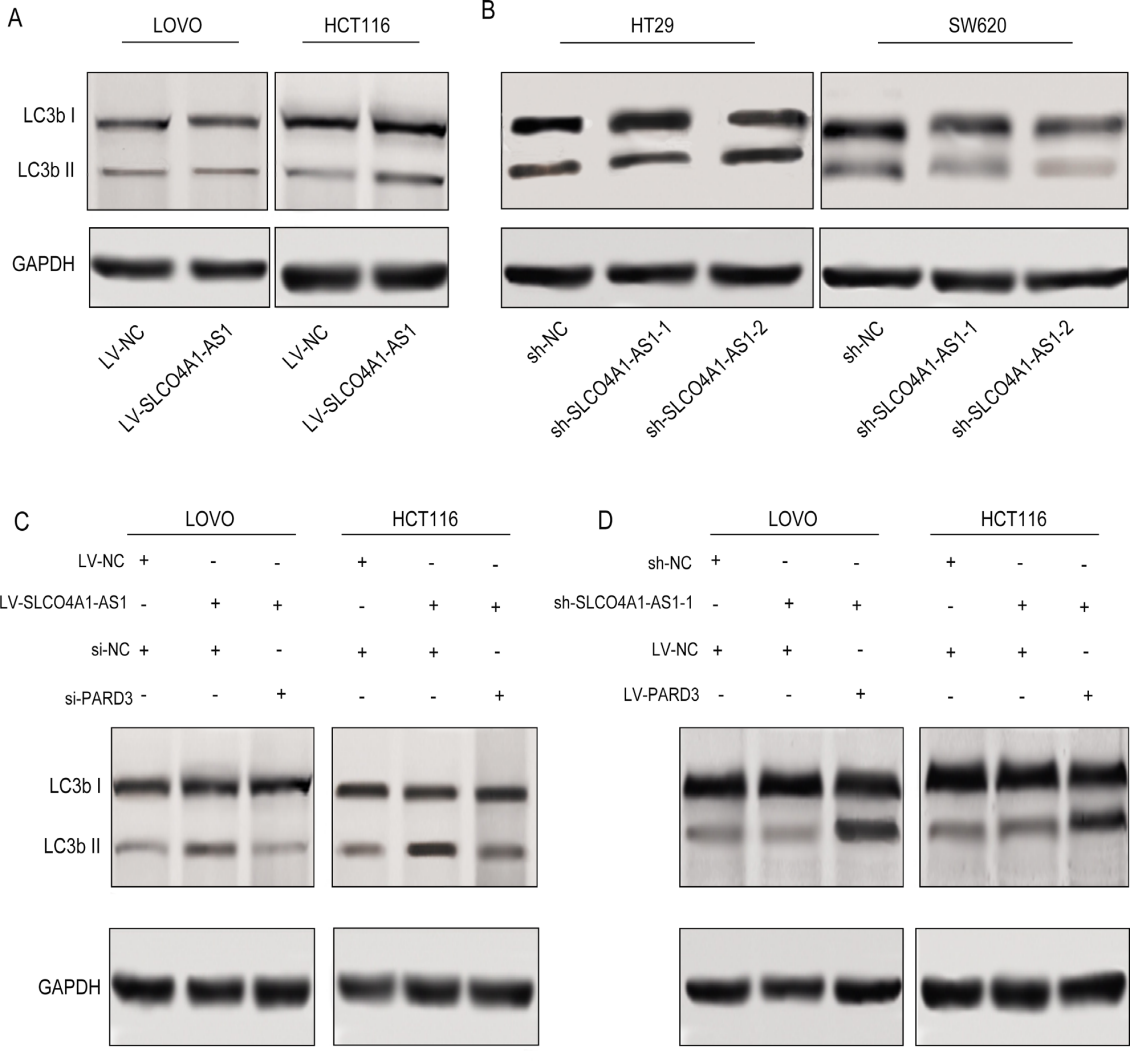
**SLCO4A1-AS1 regulated autophagy via PARD3.** (**A**) LC3b after up- or down-regulation of SLCO4A1-AS1 in LOVO and HCT116 cells by Western blot analysis. (**B**) LC3b II expression was suppressed after SLCO4A1-AS1 knockdown; (**C**) LC3b I/II expression in SLCO4A1-AS1 stably-expressed LOVO and HCT116 cells after PARD3 depletion. (**D**) LC3b II expression in SLCO4A1-AS1-knowndown HT29 and SW620 cells was rescued after PARD3 restoration

As shown in Figure 5A, SLCO4A1-AS1 overexpression significantly induced autophagy, as shown by the increase of LC3b II expression, whereas, SLCO4A1-AS1 down-regulation significantly inhibited the autophagy, as revealed by decreased LC3b II expression (Figure 5B). The loss of PARD3 significantly weakened the role of SLCO4A1-AS1 in autophagy (Figure 5C), while overexpression of PARD3 significantly remedied the defect of SLCO4A1-AS1 (Figure 5D), indicating that SLCO4A1-AS1 induces autophagy by up-regulating PARD3 expression.

### Effect of SLCO4A1-AS1 on the tumor growth of CRC

**Figure 6:**
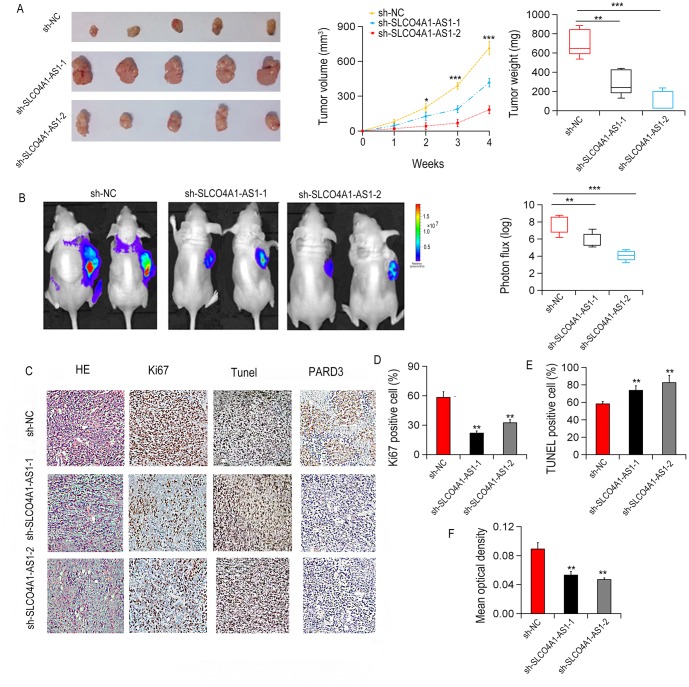
**SLCO4A1-AS1 knockdown inhibited CRC growth in xenograft nude mice.** (**A**) Tumor weight and volumes in xenograft nude mice (n = 5) after subcutaneous injection withSW620-sh-SLCO4A1-AS1 or SW620-sh-NC cells. (**B**) Representative images and photon influx of xenograft tumors in mice after subcutaneous injection with SW620-SLCO4A1-AS1 or SW620-sh-NC cells. (**C–F**) Representative images of H&E staining, Ki67, TUNEL, and PARD3. *P < 0.05, **P < 0.01 and ***P < 0.001

We established xenograft models of nude mice with SLCO4A1-AS1 knockout and wild type SW620 cells to investigate the effect of SLCO4A1-AS1 on tumor growth. SLCO4A1-AS1shRNAs or control shRNA were stably transfected into SW620 cells (SW620-sh-SLCO4A1-AS1-1, SW620-sh-SLCO4A1-AS1-2 and SW620-sh-NC) mediated by lentivirus vector. The result showed that the growth potential of SW620-sh-SLCO4A1-AS1 group was significantly lower than that of SW620-sh-NC group (Figure 6A). Down-regulation of SLCO4A1-AS1 significantly inhibited the overall tumor growth (Figure 6B). Compared withSW620-sh-NC cells, the staining of PARD3 or Ki67 of SW620 sh-SLCO4A1-AS1 cells of xenografts markedly decreased. The number of Terminal labeled positive cells increased when SLCO4A1-AS1 was also inhibited (Figure 6C–6F). The findings indicated that inhibition of SLCO4A1-AS1 weakened the growth of CRC, which is consistent with the findings observed *in vitro*.

### SLCO4A1-AS1 regulated PARD3 expression via serving as a sponge molecule of miR-508-3p

**Figure 7:**
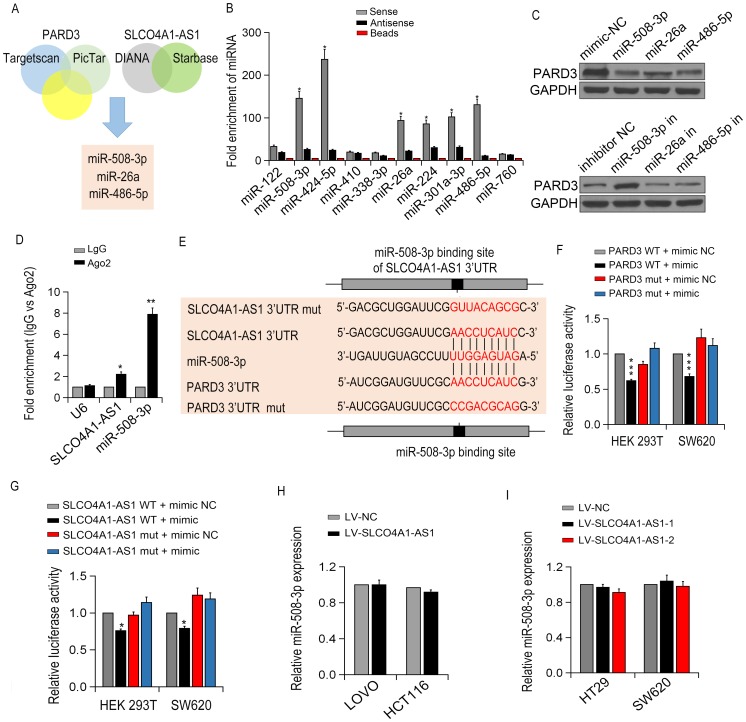
**SLCO4A1-AS1 regulated PARD3 expression by sponging miR-508-3p.** (**A**) The diagram illustrated the predicted and mutant 3’-UTR of PARD3; (**B**) The targeted miRNA levels were measured by qRT-PCR in SW620 cells; (**C**) PARD3 expression in SW620 cells after transfection with specific miRNA mimics or inhibitor (miR-508-3p, miR-26a,miR-486-5p) detected by Western blot. (**D**) Enrichment of SLCO4A1-AS1 and miR-508-3p by using AGO2 antibody in SW620 cells by RIP assay. (**E**) miR-508-3p binding sites in SLCO4A1-AS1 3’-UTR region were predicted by TargetScan; (**F**, **G**) Luciferase activity of HEK293T and SW620 cells after co-transfection with PARD3-3’UTR-WT (or Mut) and miR-508-3p or SLCO4A1-AS-3’UTR-WT (or Mut)and miR-508-3p detected by dual-luciferase assays; (**H**, **I**) miR-508-3p expressions with up- or down-regulation of SLCO4A1-AS1 expression in CRC cells by qRT-PCR.

To determine whether SLCO4A1-AS1 serves as a ceRNA, we use online bioinformatics analysis (miRanda, PicTar and TargetScan) to predict the potential miRNA binding sites. Then, RNA pull-down tests determined the involved miRNAs interacted with SLCO4A1-AS1, and miR-508-3p, miR-26a and miR-486-5p were identified to interact with SLCO4A1-AS1 (Figure 7B). We then detected the expression of PARD3 in SW620 cells after up- or down-regulating miRNA expression. Interestingly, among these miRNAs, the expression of endogenous PARD3 was significantly down-regulated by miRNA-508-3p (Figure 7C). Besides, the specific interaction of SLCO4A1-AS1 and miR-508-3p in SW620 cells was confirmed by RIP assay (Figure 7D).

As shown in Figure 7E–7F, luciferase reporter gene assay showed that the expression of WT 3'UTR of PARD3 was decreased with the presence of miR-508-3p, whilePARD33'UTR Mut did not respond much to miR-508-3p. MTS and colony formation assays showed that overexpression of miR-508-3p in LOVO cells inhibited cell proliferation, which was restored by the up-regulation of SLCO4A1-AS1. Luciferase activity assay demonstrated that ectopic expression of SLCO4A1-AS1 restrained the inhibitory effect of miR-508-3p on cell proliferation and apoptosis, indicating that SLCO4A1-AS1 bound directly to miR-508-3p (Figure 7G). However, no significant change in the miR-508-3p level was observed after overexpression or knockdown of SLCO4A1-AS1 (Figure 7H–7I). Taken together, our data indicated that SLCO4A1-AS1 regulates PARD3 expression via acting as a sponge molecule of miR-508-3p.

## DISCUSSION

In this study, we found thatSLCO4A1-AS1 induces protective autophagy and promotes CRC cell growth both *in vitro* and *in vivo*. Specifically, SLCO4A1-AS1 served as ceRNA via competing with endogenous miR-508-3p, thereby enhancing the expression of PARD3. These findings revealed a potential role of SLCO4A1-AS1 in regulation of autophagy and proliferation in CRC.

It was recently reported that abnormal expression of lncRNAs, such as KAT7, MALAT1, TINCR and HOXD-AS1, plays a significant role in the occurrence and development of CRC [13–16]. In this study, we found that SLCO4A1-AS1 levels in CRC tissues were significantly higher than those in corresponding adjacent control tissues, which were consistent with the findings in microarray gene expression profile [17]. Similarly, relative to the normal intestinal epithelium cell line NCM460, the expression of SLCO4A1-AS1 was significantly higher in CRC cell lines. Moreover, the higher level of SLCO4A1-AS1 was observed in the more advanced the clinical stage, indicating that SLCO4A1-AS1 plays a carcinogenic role in CRC.

Autophagy is a highly conservative process of cell self-degradation, which plays a vital role under stress conditions and cell survival [18]. Various cancer types, including CRC, take autophagy as a nutritional source during tumor growth [19]. PARD3 is downstream of mTORC1 and AMPK to sense amino acids and energy signaling and thus triggers the initiation of autophagy [20]. PARD3 is up-regulated in many types of tumors, which is related to the progress of tumors and the chemotherapy resistance [21]. Our results illustrated that the expression of PARD3 protein was positively correlated with SLCO4A1-AS1 in human CRC tissues. Furthermore, functional gain and loss experiments demonstrated that SLCO4A1-AS1 induces autophagy through enhancing PARD3 protein expression. Therefore, our study confirmed that the increase of PARD3 level is due to the oncogene SLCO4A1-AS1, which provides a potential mechanism for the up-regulation of PARD3 in CRC.

Previous studies indicated that SLCO4A1-AS1 interacted with Nucleolin, c-Myc or NOP2 to regulate proliferation [22]. Recently, it has been shown that some lncRNAs serve as “sponges” to regulate specific miRNAs functions by binding with them. For instance, in gastric cancer lncRNA HOTAIR promotes the growth and invasion of cancer cells via enhancing the expression of HER2 through miR-331-3p [23]. In hepatocellular carcinoma, LncRNA HULC directly binding to miR-372 to regulate its expression and the activity of PRKACB. Our study confirmed that SLCO4A1-AS1 also functioned as a sponge of miR-508-3p. Furthermore, we found that the down-regulation of SLCO4A1-AS1 inhibited expression of the target gene PARD3 of miR-508-3p, while up-regulation of SLCO4A1-AS1 inhibited the function of miR-508-3p in CRC. Therefore, SLCO4A1-AS1 serves as a molecular sponge of miR-508-3p, which modulates the autophagy and proliferation of CRC cells by upregulatingPARD3 expression. Our findings illustrate a potential mechanism of how SLCO4A1-AS1 regulates oncogenic status. However, our study also has limitations regarding the small sample size and the generalizability of the findings in these experimental models to human disease in vivo. Future investigations are needed to highlight the functional role of SLCO4A1-AS1 in larger CRC cohorts.

## MATERIALS AND METHODS

### Clinical samples

A total of 23 CRC specimens and adjacent non-tumor tissues were obtained from patients undergoing surgical resection surgery at the First Affiliated Hospital and College of Clinical Medicine of Henan University of Science and Technology between January 2015 and December 2016. All patients who had not received chemotherapy or radiotherapy prior to the operation were enrolled in the study. Clinicopathological data including age, gender, TNM stage, and lymph node metastasis, were noted in Supplementary Table 1. All subjects gave written informed consent in accordance with the Declaration of Helsinki principles. The protocol applied in this study was approved by the Ethics Committee of the First Affiliated Hospital and College of Clinical Medicine of Henan University of Science and Technology.

### Cell lines

CRC cell lines (DLD-1, SW480, HT29, SW620, HCT116, LOVO, RKO) and normal intestinal epithelial cell lines NCM460 were purchased from ATCC (MA, VA, USA), which were cultured in RPMI 1640 medium (Invitrogen, Carlsbad, CA, USA) with 10% fetal bovine serum (FBS). NCM460 cells were incubated in serum-free medium (Invitrogen) (containing 1% penicillin/streptomycin, 0.2 ng/mL recombinant endothelial growth factors. The incubator was humidified and maintained at a temperature of 37°C, with 5% CO2.

### qRT-PCR

Trizol reagent (Cat.: 15596026, Life Technologies, MA, USA) was applied to isolate the total RNA from tissues or cultured cells in accordance with the manufacturer’s instructions. Superscript III reverse transcription kit was used to obtain 20 ug/L RNA with a final volume of 10μl (Cat.: 18080200, Life Technologies). Real-time PCR was performed with gene-specific primers in the presence of SYBR Premix Ex Taq (Cat.: RR420A, TaKaRa, Japan). qPCR amplification conditions were as follows: 95°C for 10 min, 94°C for 30 s, 60°C for 15 s, and 72°C for 30 s for 35 cycles in Real-Time PCR Instrument (Applied Biosystems, USA). Relative mRNA expression level was calculated using the formula2-^ΔΔCt^.

### Subcellular grading

The PARIS Kit (Cat.: AM1921, Life Technologies) was operated in compliance with the instruction to isolate the cytoplasmic and nuclear RNA to determine the cellular localization of SLCO4A1-AS1. GAPDH and U6 were taken as internal reference genes of the cytoplasm and nucleus respectively in qRT-PCR.

### Plasmid construction

Two small hairpin RNA (shRNA) sequences, namely LV-shSLCO4A1-AS1-1 and LV-shSLCO4A1-AS1-2 and the control shRNA (LV-shNC) were constructed by Huayueyang Biotechnology (Beijing, China). The knockdown efficiency was detected with qRT-PCR. To obtain SLCO4A1-AS1 overexpression vector (LV-SLCO4A1-AS1) was synthesized and subcloned into pGLV3/H1/GFP/Puro plasmid (GenePharma). The negative control was LV-NC.

### Cell growth assay

Cell proliferation assay was performed with the Cell Titer 96 Non-Radioactive Proliferation Assay Kit (Promega, Madison, WI, USA). 1.5 x 10^3^ cells were cultured onto 96-well plates. Cell-Light TMEdU DNA Cell Proliferation Kit (Cat.: C10310, RiboBio, Guangzhou, China) were applied to perform immunofluorescent staining with 5-ethynol-2'-deoxyuridine (EdU). The collected cells were isolated and seeded into 6-well plates (1.5 x 103 cells per well) to conduct colony formation assay. The medium was changed every 3 days. The trials were repeated in triplicate.

### Flow cytometry analysis

Flow cytometry analysis was performed to detect the cell cycle and apoptosis. Flow cytometric analysis was conducted as previously described [24].

### Western blotting

Cell lysates were prepared with PLC lysis buffer and were applied onto 10% or 15% SDS-PAGE. After running, the proteins in the gel were transferred onto PVDF membranes. The ECL chemiluminescent reagent (Cat.: RPN2109, GE Healthcare, USA) was used to detect chemical signals. Antibodies including PARD3 (Cat.: ab191204), LC3b (Cat.: ab192890) and GAPDH (Cat.: ab181602) antibodies were purchased from Abcam (Cambridge, MA, USA).

### *In situ* hybridization

The thickness of the tissue section of CRC was 4 μm, which was treated with 20 ug/mL protease K at 37 °C for 8 min post dewaxing. The section was pre-hybridized with ISH buffer (Exiqon), followed by hybridization with digoxigenin labelled probe for 40 min at 45 °C. Hybridized sections were incubated overnight with digoxigenin antibody (Roche Diagnostics IN) at 4°C and then were stained with nitroblue tetrazole/5-bromo-4-chloro-3- indolyl phosphate.

### Immunohistochemistry

Immunohistochemistry was performed as previously described [25]. Briefly, samples were paraffin embedded and cut into 4μm sections. Sections were placed on slides coated with polylysine. Then slides were deparaffinised in xylene and rehydrated with graded alcohol. Antigen retrieval was performed with heated citrate buffer (pH 6). After blocking with 10% goat serum, samples were incubated with primary antibodies, against Ki67 (ab15580, Abcam, Cambridge, MA, USA), PARD3 (ab191204, Abcam), and horseradish peroxidase coupling IgG. The protein was observed in situ with Super Sensitive Link-Label IHC Detection System (Cat.: LA000-ULE, BioGenex, Fremont, CA, USA).

### Autophagy flux activity detection

Cells were seeded on 6-well plates at a density of 2 x 10^4^ cells/well and were cultured in DMEM medium. The mRFP-GFP-LC3 adenovirus vector was used for detection of autophagy activity, which was purchased from Gene Chem (Shanghai, China). Adenovirus infection trial was carried out according to the instruction. The autophagy flux activity was detected under confocal microscopy (Zeiss, Oberkochen, Germany). In details, the autophagic flux was evaluated by calculating GFP and mRFP point numbers.

### Luciferase reporter assay

Human SLCO4A1-AS1 3'UTR luciferase reporter gene SLCO4A1-AS1-wt (containing a miR-508-3p response element) and SLCO4A1-AS1-mut (containing a mutant MREs-939-5p) was constructed via cloning the SLCO4A1-AS1 mRNA 3'UTR sequence into the downstream of luciferase reporter gene vector (Cat.: E1751, Promega). Wild-type PARD3 (PARD3-wt) and the mutant derivate (PARD3-mut) lack of miR-508-3p binding site were cloned into the downstream of luciferase gene coding sequence. Lipofectamine 2000 (Cat.: 11668027, Invitrogen) was applied to co-transfect HEK293T and SW620 cells with the luciferase reporter plasmid with miR-508-3p mimics or miR-NC. All trials were repeated for three times.

### Animal model

Four-five weeks old female athymic BALB/c nude mice raised under sterile conditions were used for construction of mouse models. To generate xenograft mice, 1×10^7^ SW620 cells transfected LV-shSLCO4A1-AS1-1, LV-shSLCO4A1-AS1-2 or LV-shNC were subcutaneously injected into nude mice. Xenograft mouse tumors were monitored through In-Vivo fluorescent imaging system (IVIS), and the tumor size and weight of xenograft mice were measured after 4 weeks transfection. Tumor growth in xenograft mice was observed under IVIS system. The tumor volume was calculated as length x width ^2^ x 0.5. The study was performed at the First Affiliated Hospital and College of Clinical Medicine of Henan University of Science and Technology in strict accordance with the guidelines of the Care and Use of Laboratory Animals, and the protocol was approved by the Ethics Committee of Animal Experiments at the First Affiliated Hospital and College of Clinical Medicine of Henan University of Science and Technology.

### RNA pull-down test

The RNA pull-down test was performed as described before [26]. SLCO4A1-AS-sense and SLCO4A-AS1-antisense were transcribed from pGEM-T-SLCO4A1-AS1 vector *in vitro* (Biotin RNA label combination and T7 RNA polymerase (Cat.: EP0111, Thermo Fisher Science) label biotin were used), which was treated with DNase I (Cat.: 18047019, Thermo Fisher Science) without RNAs and then purified with RNeasy Mini kit (Cat.: 74104, Qiagen, Valencia, CA, USA). The RNA mixture was bound to the microspheres and used for qRT-PCR analysis.

### Statistical analysis

SPSS 18.0 software (SPSS, Chicago, IL, USA) was applied for statistical analysis. All numerical data were shown based on multiple samples mean ± standard deviation. Differences among the groups were analyzed using one-way analysis of variance (ANOVA) followed by Tukey’s test for multiple comparisons. The correlation of SLCO4A1-AS1, PARD3 and miR-508-3p was analyzed with Pearson rank correlation analysis. A p-value < 0.05 was considered as statistically significant.

## CONCLUSIONS

In summary, our study revealed that SLCO4A1-AS1 was significantly upregulated in CRC tissues. SLCO4A1-AS1 promotes autophagy and CRC cell proliferation. More importantly, we demonstrated for the first time that SLCO4A1-AS1 is the sponge of miR-508-3p in the up-regulation of PARD3. Our study uncovered that SLCO4A1-AS1/miR-508-3p/PARD3/autophagy pathway might provide novel targets for CRC therapy.

## Supplementary Material

Supplementary Table 1
