# Supplementary material for: LncRNA SLCO4A1-AS1 promotes colorectal cancer cell proliferation by enhancing autophagy via miR-508-3p/PARD3 axis
Source: Aging (Albany NY). 2019 Jul 16;11(14):4876–89. doi: 10.18632/aging.102081 (PMC6682525; doi:10.18632/aging.102081)
Supplement: Supplementary Table 1 [file aging-11-102081-s001.pdf]

SUPPLEMENTARY TABLE

Supplementary Table 1. Clinical pathological characteristics.

NO	Age	Gender	TNM stage	Lymph node metastasis
1	45	Female	IIA	N0
2	70	Male	IV	N1
3	69	Male	IIIB	N2
4	70	Female	IIA	N0
5	58	Male	I	N0
6	70	Female	IIB	N1
7	77	Male	IIA	N0
8	82	Male	IIIC	N 2
9	80	Female	IIIB	N 0
10	45	Male	IIA	N 0
11	70	Male	IIIC	N 1
12	69	Male	IIIB	N 2
13	72	Male	IIA	N 0
14	58	Male	IIA	N 0
15	70	Female	IIIC	N 0
16	46	Male	IIIB	N 1
17	70	Male	IIA	N 0
18	69	Male	I	N 1
19	73	Male	IIB	N 0
20	58	Male	IIA	N 0
21	70	Female	IIIB	N 1
22	77	Male	I	N 0
23	82	Female	I	N 0
